# Increasing Pain Sensation Eliminates the Inhibitory Effect of Depression on Evoked Pain in Rats

**DOI:** 10.3389/fnbeh.2016.00183

**Published:** 2016-09-28

**Authors:** Ning Wang, Sheng-Guang Li, Xiao-Xiao Lin, Yuan-Lin Su, Wei-Jing Qi, Jin-Yan Wang, Fei Luo

**Affiliations:** ^1^CAS Key Laboratory of Mental Health, Institute of Psychology, Chinese Academy of SciencesBeijing, China; ^2^University of Chinese Academy of SciencesBeijing, China

**Keywords:** unpredictable chronic mild stress, pain, serotonin, descending inhibition, avoidance motivation

## Abstract

Although previous studies have suggested that depression may be associated with inhibition of evoked pain but facilitation of spontaneous pain, the mechanisms underlying these relationships are unclear. The present study investigated whether the difference between evoked and spontaneous pain on sensory (descending inhibition) and affective (avoidance motivation) components contributes to the divergent effects of depression on them. Depressive-like behavior was produced in male Wistar rats by unpredictable chronic mild stress (UCMS). Tone-laser conditioning and formalin-induced conditioned place avoidance (F-CPA) were used to explore avoidance motivation in evoked and spontaneous pain, respectively. Behavioral pharmacology experiments were conducted to examine descending inhibition of both evoked (thermal stimulation) and spontaneous pain behavior (formalin pain). The results revealed that the inhibitory effect of depression on evoked pain was eliminated following repeated thermal stimuli. Avoidance behavior in the tone-laser conditioning task was reduced in UCMS rats, relative to controls. However, avoidance motivation for formalin pain in the UCMS group was similar to controls. 5-HT_1A_ receptor antagonism interfered with inhibition of pain responses over time. The present study demonstrated that the inhibitory effect of depression on evoked pain dissipates with increased nociception and that the sensory-discriminative and affective-motivational components of pain are jointly involved in the divergent effects of depression on pain.

## Introduction

Comorbidity of pain and depression is common (Ohayon and Schatzberg, 2003; Poole et al., 2009; Fitzgibbon et al., 2016), with prior reports indicating that average of 65% patients with depression suffer from pain (Bair et al., 2003). Clinical studies have indicated that patients in a depressed state have elevated pain perception (Jaracz et al., 2016). Meanwhile, laboratory studies have documented reduced sensitivity to thermal (Bär et al., 2005; Boettger et al., 2013), electrical (Bär et al., 2005; Marsala et al., 2015), or mechanical (Graff-Guerrero et al., 2008) forms of pain in patients with depression. This contradiction was somewhat perplexing until Shi et al. (2010a,b) discovered that depression has opposite effects on spontaneous vs. evoked pain in animal models (Wang et al., 2010). They demonstrated that depression inhibited stimulus-evoked pain responses but facilitated spontaneous (stimulus-independent) pain behaviors, even in animals experiencing concurrent spontaneous and evoked pain (Su et al., 2010). Hence, new questions emerged regarding how depression could have opposite effects on different forms of pain.

Differences in the intrinsic characteristics of stimulus-evoked and spontaneous pain may explain the aforementioned seemingly contradictory effects. With respect to external behaviors, spontaneous pain, which is persistent and unavoidable, induces stronger nocifensive behaviors (e.g., continuous licking; Tjølsen et al., 1992) than does brief thermal or electrical stimulation, which induce only simple nociceptive responses (e.g., paw withdrawal). Neurologically, spontaneous pain involves much more supraspinal processing than does evoked pain, including activation of the endogenous descending inhibitory system (Omote et al., 1998) and involvement of emotional processing in cortical and subcortical circuits (Cao et al., 2009, 2014; Jiang et al., 2014). Evoked pain may induce less pain-related emotion because it is transient and avoidable (Lumley et al., 2011). Indeed, the amygdale, a key site of emotional processing, has been shown to be activated by persistent pain, but not by acute experimental pain (Kulkarni et al., 2005).

Depression and pain share common neuroanatomical pathways and neurobiological substrates (Gambassi, 2009; Ledermann et al., 2016). Pathways altered by depression may interact with multiple components of pain processing. Studies have demonstrated that the serotoergic dysfunction plays an important role in the development of depression (Kaufman et al., 2016). Meanwhile, the sensory-discriminative dimension of pain is modulated by endogenous descending inhibitory serotonergic pathway (Ossipov et al., 2014). Therefore, the deletion of serotonin may decrease the modulatory effect of this descending pain system (Bair et al., 2003). Although amotivation and anhedonia are core symptoms of depression (Ågren and Reibring, 1994; Nestler and Carlezon, 2006; Treadway and Zald, 2011), the effect of depression on the affective-motivational dimension of pain is unclear. Our previous electrophysiological studies showed that depression altered processing patterns and information interactions in the medial (affective) pain pathway, which suggested that a depressive state may influence pain affection (Wang et al., 2013). However, direct evidence is needed to clarify how depression influences the pain-avoidance motivation.

Based on the aforementioned findings and implications, we hypothesized that differences in the sensory-discriminative dimension (i.e., pain intensity) and the affective-motivational dimension (i.e., avoidance motivation) between evoked and spontaneous pain may account for the divergent effects of depression on evoked vs. spontaneous pain. To test this hypothesis, we conducted a series of behavioral experiments in rats: (1) Multiply repeated thermal stimulations were used, in order to produce a higher level of pain intensity. We expected to observe a transition from an inhibitory to facilitatory effect of depression modulation on evoked pain; (2) Using tone-laser conditioning paradigm and formalin-induced conditioned place avoidance (F-CPA) paradigm, we established a relationship between evoked/spontaneous pain and tone/context and examined the avoidance motivation for both conditions in rats subjected to unpredictable chronic mild stress (UCMS). The behavioral reactions induced by conditioned tone could reflect the avoidance motivation of evoked pain (Li et al., 2012), and the conditioned place avoidance behavior was used to investigate the response motivated by ongoing pain (Johansen et al., 2001; Navratilova and Porreca, 2014). We predicted that the amotivation symptom of depression may lead to decreased pain-avoidance motivation, thus contributing to the inhibitory effect of depression on evoked pain; and (3) Given that monoamine neurotransmitters are shared by pain and depression (Han and Pae, 2015; Kaufman et al., 2016), we hypothesized that alteration of nociceptive sensitivity in the depressive state may be mediated, at least in part, via serotonin (5-HT) receptors. To test this hypothesis, we examined the effects of 5-HT_1A_ receptor antagonism on behaviors associated with repeated thermal evoked pain and with ongoing spontaneous pain.

## Materials and Methods

### Animals

A total number of 151 male Wistar rats (220–250 g) from the Laboratory Animal Center of the Academy of Military Medical Science were used in this study. All rats were housed individually in specific pathogen free conditions. Food and water were available free. The UCMS-exposed and control groups were kept in separated rooms so that their environments could be manipulated independently. The rooms were maintained at 22 ± 2°C with a standard 12-h light-dark cycle (lights on at 07:00 am). Testing was performed during the light cycle. Rats were acclimated for 1 week before the experiments, and they were handled gently 3~5 min per day by the experimenter. All experimental procedures were approved by the Institutional Review Board of the Institute of Psychology, Chinese Academy of Sciences (No. A12013).

### Experimental Design

This study includes three sets of experiments. In the first experimental set, 12 rats were randomly and equally assigned to UCMS and control groups. We examined the nociceptive responsivity (thermal nociceptive thresholds for paw withdrawal) of animals subjected to UCMS relative to no-UCMS controls with the aim of probing whether the divergent effect of depression on pain may be related to nociceptive sensitivity.

In the second experimental set, we examined avoidance behavior in two distinct conditioning paradigms, i.e., tone-laser conditioning and F-CPA, using UCMS vs. control rats: the tone-elicited response is thought to reflect motivation to avoid evoked pain, whereas the behavioral expression in F-CPA task indicates motivation to avoid spontaneous/ongoing pain. In the tone-laser conditioning, 23 rats were randomly divided into UCMS (*n* = 13) and control groups (*n* = 10). In the F-CPA task, 68 rats were randomly allocated into UCMS (*n* = 38) and control groups (*n* = 30). There were 18 rats in UCMS group and 14 rats in control group received 2.5% formalin injection and the other rats were injected with 5% formalin.

Finally, in the third experimental set, we examined the effects of systemic 5-HT antagonism on nociceptive responsivity in both evoked pain (from thermal stimulus) and spontaneous pain (from formalin injection) paradigms in behaviorally naïve (no UCMS) animals. Forty-eight naïve rats were randomly and equally divided into treatment and control groups. In each group, half rats were used in thermal pain test and the other half in formalin test. In the treatment group, 5-HT_1A_ receptor antagonist WAY-100635 (0.3 mg/kg, Sigma-Aldrich) was injected subcutaneously 30 min before pain testing. The control group was injected with the same volume of saline.

### UCMS Procedure

The UCMS animal model is a classic depression model (Willner et al., 1987). As described in our previous studies (Shi et al., 2010b; Wang et al., 2013), the following stressors were presented in a pseudo-random order during each weekly cycle: exposure to a hot room (40°C, two 30-min periods), water deprivation (22-h and 40-h periods), empty water bottle (one 1-h period, exposure to empty water bottle immediately after one 40-h period of water deprivation), cage tilt (45°, 8-h and 16-h periods), intermittent white noise (75 dB, 2-h and 5-h periods), novel odor (two 16-h periods), overnight illumination (two 16-h periods), exposure to a cold room (10°C, two 30-min periods), soiled bedding (two 16-h periods), food deprivation (20-h and 22-h periods), restricted access to food (one 3-h period, two small pieces of pellet in each cage following one 20-h period of food deprivation), and strobe light (7-h and 8-h periods). The UCMS exposure period was 4 or 6 weeks.

### Depressive-Like Behavior

Sucrose preference tests were performed weekly before and during the UCMS exposure period. This test was conducted in each rat’s home cage for 1 h, following a 22-h period of food and water deprivation. Two bottles, one containing sucrose solution (1%) and the other containing water, were presented simultaneously to each rat. Sucrose preference was calculated according to the formula:

$$\begin{array}{l} \%\;\text{sucrose}\;\text{preference}\;=\\ \frac{\text{sucrose}\;\text{solution}\;\text{consumption}}{\left(\text{sucrose}\;\text{solution}\;\text{consumption}\;\text{+water}\;\text{consumption}\right)}\;\times \;100 \end{array}$$

### Evoked Pain Test

Radiant heat was used to induce thermal evoked pain. The apparatus and test for thermal evoked pain were the same as described in our previous studies (Shi et al., 2010b; Su et al., 2010). Briefly, the animals were placed in a Plexiglas chamber with a glass floor beneath which a radiant heat apparatus (10 W projector bulb) was situated. Light was focused on the plantar surface of a hindpaw. Paw withdrawal latency (PWL) induced by the thermal stimulation was adopted as a measure of pain thresholds. A cutoff time of 22 s was imposed to prevent tissue damage. The inter-stimulus interval was 40~60 s.

In the formalin test, a subcutaneous injection of 5% formalin (50 μl) was delivered into the plantar surface of a hindpaw. The rat was returned to the testing chamber immediately. A computer-based video recording system was used to record each animal’s behavior for 60 min. Time spent licking the affected paw was calculated for each 5 min interval within the recording period.

### Tone-Laser Conditioning

#### Apparatus and Conditioning

A custom-designed Plexiglas chamber (22 × 22 × 30 cm) was used (Li et al., 2012). The chamber had holes (3-mm diameter at 3-mm intervals) in the bottom and a speaker on its back wall. Tones (80 dB, 2900 Hz, 100-ms duration) from the speaker were used as the conditioned stimuli (CS). A noxious laser radiation beam in the infrared spectrum (10.6-μm wave length, 20 ms pulse width) generated from a surgical CO^2^ laser stimulator (Model DM-300, Changchun Institute of Optics, Fine Mechanics and Physics, Chinese Academy of Science) served as the unconditioned stimulus (US). The laser beam projection was guided by a helium laser that produced a red light spot, it was applied to the plantar surface of the rat hindpaw with a 1-mm diameter. The location of the stimulation site was varied to avoid skin damage, sensitization, or habitation. Between rats, the chamber was cleaned with 75% alcohol. To ensure an equivalent sensation level, the laser power was set to the intensity that induced a 70% withdrawal response (7 out of 10 attempts) for each rat. In each pairing trial, the tone cue (CS) was presented 5 s before the laser stimulation (US). The average inter-trial interval was 75 s (range 60~90 s). The tone-laser conditioning included three phases: baseline (10 trials of tone alone), conditioning (30 trials of tone-laser pairing), and extinction (30 trials of tone alone).

#### Behavioral Assessment

Stimulus-elicited behaviors (Fan et al., 1995; Li et al., 2012) were scored as follows: 0, immobility; 1, head turning (shaking or elevating the head); 2, flinching (a small abrupt jerking body movement); 3, withdrawal (retracted of the paw away from the stimulus) or licking (paw retraction flowed by licking of that paw); and 4, body movement (body turning and running). The rats were videotaped throughout the experiment. Only the maximum score was recorded within each trial. The behavioral responses were assessed by cumulative scores for every five successive trials.

### F-CPA

The F-CPA experiments were conducted in a plastic chamber (75 × 30 × 30 cm, length × width × height) with three in-line compartments. Formalin was paired with one of two “conditioning” (end) compartments (30 × 30 × 30 cm). The third (middle) compartment (15 × 30 × 30 cm) was “neutral” compartment. Distinct tactile and visual cues characterized the three compartments. One conditioning compartment had a grid floor with vertical stripes on the walls, whereas the other had a holey floor with horizontal stripes. The neutral compartment had walls that were uniform in color (black) and a plain floor.

The CPA procedure included three phases (Jiang et al., 2014): baseline (pre-training), conditioning (training) and testing. In the baseline phase (days 1 and 2), rats were allowed to explore the three compartments freely for 900 s. The time spent in each compartment and locomotor distance in the whole chamber were measured automatically (MacroAmbition S&T Development, Beijing, China) and averaged across the two baseline days. Baseline place preference testing confirmed that the animals showed no initial preferences for either of the conditioning compartments before conditioning. The conditioning phase included and aversive conditioning trial and an unpaired trial (random between day 3 and 4). In the conditioning trial, rats were given an intraplantar injection of 2.5% or 5% formalin (50 μl) into the right hindpaw and were confined randomly to one of the conditioning compartments for 1 h. On the other conditioning day, each rat was confined in the opposite compartment for 1 h without any treatment. At the test session (day 5), each rat was given free access to the three compartments. The time spent in each compartment was recorded and measured automatically as in the baseline phase.

### Data Analysis

Statistica v10.0 (StatSoft, Inc., Tulsa, OK, USA) and GraphPad Prism 5.0 (GraphPad Software, Inc., La Jolla, CA, USA) were used for statistical analyses and graph generation. The pain thresholds, duration of licking data, data of avoidance behaviors *were analyzed* using two-way *analysis of variance* (ANOVA) followed by Duncan’s multiple comparison test. The Student’s *t*-test was employed when two groups were compared. The data were presented as the mean ± standard error of the mean (SEM). The statistical significance was set at *P* < 0.05.

## Results

### Depressive State Produces Hypoalgesia Selectively in early Repeated Thermal Stimulation Trials

**Figure 1:**
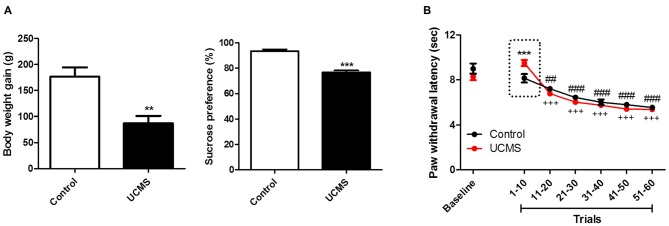
**The depressive-like behavior and nociception of repeated thermal stimuli. (A)** Body weight gain and sucrose preference in the 6th week of unpredictable chronic mild stress (UCMS). Body weight gain is the weight difference between the conclusion of 6 weeks of UCMS and immediately before beginning UCMS. **(B)** Paw withdrawal latencies (PWLs) averaged in serial 10-trial bins of repeated thermal stimuli. The dotted-box highlights the UCMS-related reduction in nociception sensitivity. **P < 0.01, ***P < 0.001 vs. control group. ^##^*P* < 0.01, ^###^*P* < 0.001 vs. trials 1~10 of control group. ^+++^*P* < 0.001 vs. trials 1~10 of the UCMS group. *N* = 6.

As shown in Figure 1A, compared to control rats not subjected to UCMS, rats subjected to UCMS for 6 weeks gained less weight (*t*_(10)_ = 3.994; *P* < 0.01) and exhibited a reduced sucrose preference (*t*_(10)_ = 7.721; *P* < 0.001), confirming that the UCMS protocol produced the expected depressive state model. Analysis of thermal nociceptive thresholds revealed that the UCMS (i.e., depressive) group had longer PWLs than the control group in the early trials, but that this difference dissipated gradually with additional trials. As shown in Figure 1B, analyzing PWLs averaged over serial trial sets (10 trials/set), the difference was expressed in the first 10 trials (*post hoc, p* < 0.001), and no longer detectable since the next 10 trials were averaged. Besides, we confirmed that PWL changed over the progression of trials (two-way ANOVA, time effect:* F*_(5,50)_ = 89.176, *P* < 0.0001; group effect: *F*_(1,50)_ = 0.088, *P* = 0.773; interaction effect: *F*_(5,50)_ = 6.765, *P* < 0.001). Both groups exhibited shorter PWLs (more rapid withdrawal) in later trial sets than in trials 1–10, and the hypoalgesia exhibited by the UCMS group, relative to the control group, in trials 1–10 was no longer evident in later trials.

### Effects of Depressive State on Pain-Related Avoidance Motivation

#### Tone-Laser Conditioning: Avoidance Motivated by Evoked Pain

**Figure 2:**
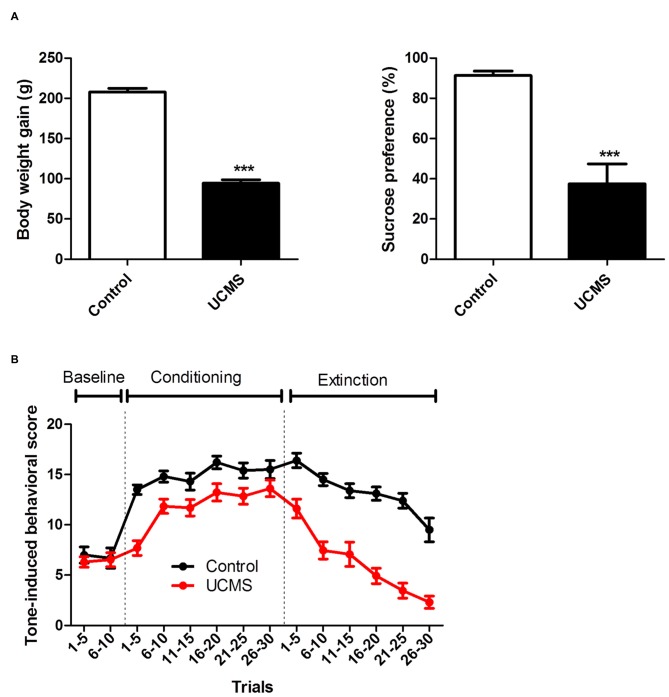
**Effect of UCMS exposure on laser-evoked nociceptive avoidance motivation. (A)** Body weight gain (relative to before UCMS) and sucrose preference after 6 weeks of UCMS. **(B)** Tone-induced behavior before conditioning (baseline), during tone-laser conditioning and at the test session (extinction). ***P < 0.001 vs. control group. *N* = 10~13.

Significant depressive like characteristics, namely less weight gain (*t*_(21)_ = 17.84; *P* < 0.001) and a reduced sucrose preference (*t*_(21)_ = 4.697; *P* < 0.001) relative to controls, were again confirmed after 6 weeks of UCMS (Figure 2A). As shown in Figure 2B, no difference was observed between the two groups during the baseline session. During the training session, UCMS rats exhibited less avoidance in response to the tone cue than control rats, with the difference being particularly pronounced in the first five trials (Two-way ANOVA, time effect: *F*_(5,105)_ = 11.114, *P* < 0.0001; group effect: *F*_(1,105)_ = 19.135, *P* < 0.001; interaction effect: *F*_(5,105)_ = 2.243, *P* = 0.055). During the extinction session (CS presented without US), avoidance behaviors extinguished more rapidly in the UCMS group than in the control group (Two-way ANOVA, time effect: *F*_(5,105)_ = 38.431, *P* < 0.0001; group effect: *F*_(1,105)_ = 62.374, *P* < 0.0001; interaction effect: *F*_(5,105)_ = 2.624, *P* < 0.05). The above results indicated that the pain-motivated avoidance was weakened under the conditions of long-term stress exposure.

#### F-CPA: Avoidance Motivated by Ongoing Pain

**Figure 3:**
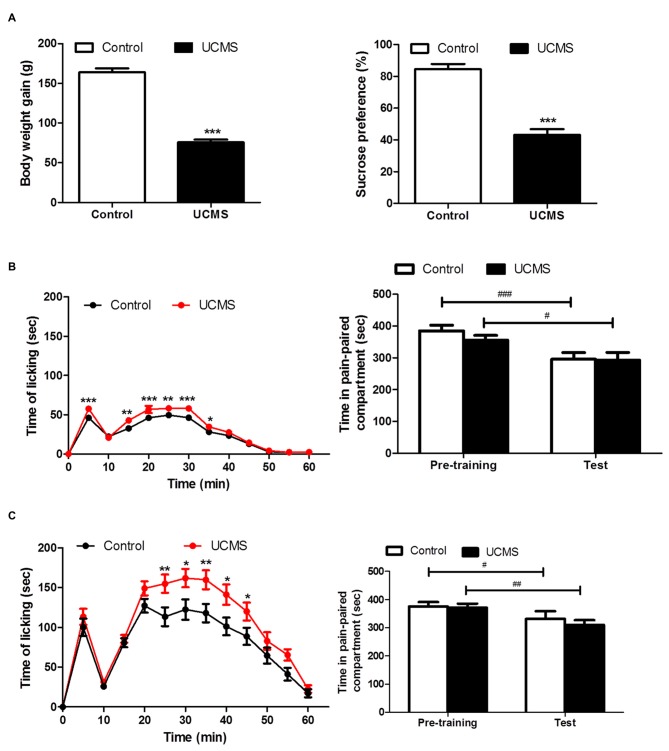
**Effect of UCMS exposure on avoidance motivation following 2.5% and 5% formalin -induced conditioned place avoidance (F-CPA) training. (A)** Body weight gain (relative to before UCMS) and sucrose preference after 4 weeks of UCMS. *N* = 30~38. **(B)** Spontaneous pain behavior after 2.5% formalin injection in training session (left) and subsequent avoidance of the conditioned compartment in the test session. *N* = 14~18. **(C)** Spontaneous pain behavior after 5% formalin injection in training session (left) and subsequent avoidance of the conditioned compartment behavior. *P < 0.05, **P < 0.01, ***P < 0.001 vs. control group. ^#^*P* < 0.05, ^##^*P* < 0.01, ^###^*P* < 0.001 vs. pre-training. *N* = 16~20.

Significant depressive-like characteristics, namely less weight gain (*t*_(66)_ = 15.02; *P* < 0.001) and a reduced sucrose preference (*t*_(66)_ = 8.289; *P* < 0.001) relative to controls, were confirmed after 4 weeks of UCMS in the UCMS group (Figure 3A). UCMS had facilitatory effect of formalin pain responsivity at both intensities (2.5% (Figure 3B) and 5% (Figure 3C) intraplantar formalin injection), as evidenced by significantly increased licking time during the F-CPA training session for the UCMS group, compared with the control group (2.5% formalin: group effect: *F*_(1,330)_ = 40.575, *P* < 0.0001; 5% formalin, group effect: *F*_(1,374)_ = 5.324, *P* < 0.05). Interestingly, for both the 2.5% formalin (Figure 3B) and 5% formalin (Figure 3C) intensities, conditioned avoidance behavior at testing was similar between the UCMS and control groups (2.5% formalin: group effect: *F*_(1,30)_ = 0.480, *P* = 0.494; 5% formalin: group effect: *F*_(1,34)_ = 0.338, *P* = 0.565). Both groups spent less time in the aversively conditioned compartment during the test session than during the pre-training session (2.5% formalin: session effect* F*_(1,30)_ = 22.22, *P* < 0.0001; interaction: *F*_(1,30)_ = 0.637, *P* = 0.431; 5% formalin: session effect* F*_(1,34)_ = 14.14, *P* < 0.001; interaction *F*_(1,34)_ = 0.382, *P* = 0.541).

### Involvement of 5-HT in Pain Response Behavior

**Figure 4:**
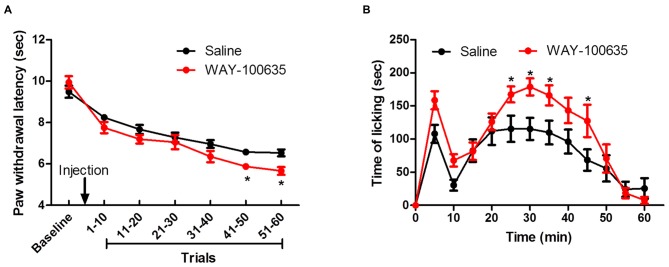
**Contribution of descending inhibitory serotonergic system on evoked and ongoing pain. (A)** Effect of 5-HT_1A_ receptor antagonist WAY-100635 on repeated thermal stimulus response. **(B)** Effect of WAY-100635 on licking behavior after 5% formalin injection. *P < 0.05 vs. saline control group. *N* = 12.

Systemic blockade of 5-HT_1A_ receptors with WAY-100635 before thermal pain testing resulted in shorter PWLs in the evoked thermal pain test, relative to saline-injected controls, with the effect becoming evident after 40 trials (Figure 4A). A two-way ANOVA demonstrated main effects of time (*F*_(6,132)_ = 77.75, *P* < 0.0001) and group (*F*_(1,132)_ = 4.772, *P* < 0.05) on PWL and a time × group interaction (*F*_(6,132)_ = 2.324, *P* < 0.05). Similarly, as shown in Figure 4B, in the spontaneous pain paradigm, time spent licking in response to a 5% formalin injection into the hindpaw was elevated by 5-HT_1A_ receptor antagonism, with the effect being particularly pronounced in the second phase of the experiment (15–60 min). A two-way ANOVA revealed main effects of time (*F*_(11,242)_ = 22.569, *P* < 0.0001) and group (*F*_(1,242)_ = 6.809, *P* < 0.05), as well as a significant time × group interaction (*F*_(11,242)_ = 2.075, *P* < 0.05).

## Discussion

In the present study, we investigated the effects of depression on both sensory and affective-motivational components of evoked vs. spontaneous pain in rats. In the first set of experiments, we found that depressed rats exhibited decreased sensitivity to noxious thermal stimuli in early (<10) trials, and the inhibitory effect vanished with further repetition of the stimulus. Thus, multiple repetitions of nociceptive stimuli are responded to differently than a single or sparse exposure to the stimulus. This result supports our hypothesis that the divergent modulatory effects of depression on pain depend on nociceptive sensitivity. Although the reverse effect (i.e., shortened PWLs) was not observed, the collapse of the inhibitory effect with stimulus repetition may suggest a tendency of transition from depressive inhibition to facilitation. In the second set of experiments, the UCMS animals exhibited reduced avoidance of both evoked and spontaneous pain. These findings support our hypothesis that depressed subjects tend to ignore mild pain while focus on intense pain and, further, suggest that the depressive state was associated with a weakened intrinsic motivation for pain avoidance. Finally, in our third experimental set, using naïve (no UCMS) rats, we found that blockade of 5-HT_1A_ receptor transmission resulted in more pronounced pain avoidance behaviors (i.e., decreased PWL to more than 40 trials thermal stimuli and more licking in response to spontaneous pain), indicating that descending inhibition of nociceptive sensitivity is mediated, at least in part, by serotonergic efferents. Thus, together, our results indicate that both sensory and affective components contribute to the divergent modulatory effects of depression on pain.

### Increasing Pain Sensation Eliminates Inhibitory Effects of Depression on Evoked Pain

Divergent effects of depression on evoked and spontaneous pain have been found in both human and rodent studies (Shi et al., 2010b; Li, 2015). Previous studies have focused mainly on the phenomenological conflict rather than clarifying differences between types of pain. Studies involving transient experimental evoked pain, in animal or human subjects, avoid primary hyperalgesia by prolonging the inter-stimulus interval or reducing the stimulus number (Iwata et al., 1994). In previous animal studies, the numbers of thermal stimuli were minimized (3~5 trials) and the inter-stimulus intervals were usually longer than 1 min (Hargreaves et al., 1988; Dirig et al., 1997; Shi et al., 2010b) to prevent central sensitization. However, spontaneous pain (modeled by the formalin test), can produce peripheral and central sensitization (Coderre and Melzack, 1992). Indeed, in the present study, we confirmed that induction of sensitization itself can lead to distinct modulatory effects of depression on pain. Our results support the notion that depression-related modulation of pain responsivity is related to the intensity of nociception and suggest that differences in subjective pain perception may contribute to the divergent effects of depression on evoked vs. spontaneous pain.

In addition, the latency to withdraw the paw depends on many factors, in particular, the local skin temperature (Vidal and Jacob, 1986). Studies have demonstrated that acute exposure to stress often lead to an increase in vasomotor tone and thus a drop in body temperature, which may influence the latency to thermal stimulation. However, it should be noted that the UCMS model represents a chronic stress, which may be different from the acute stress. For example, we have found that the plasma level of corticosterone remained unchanged after UCMS exposure (Shi et al., 2010b). It has been proposed that there is an adaptive response of HPA (hypothalamic-pituitary-adrenal) axis in the presence of prolonged high glucocorticoid concentrations (Azpiroz et al., 1999; Pignatelli et al., 2000). Therefore, chronic stress may not cause the decrease of skin temperature. In addition, our previous studies have demonstrated that UCMS exposure could increase nociceptive thresholds to both thermal and mechanical stimuli in rats, indicating that the inhibitory effect of depression on evoked pain is a robust phenomenon and may not be affected by stimulus type and local skin temperature (Shi et al., 2010a).

Serotonergic dysfunction during depression have been believed to be one of the factors that influence the perception of pain (Kundermann et al., 2009). Previous studies have shown that a deficiency in serotonin neurotransmission in the central nervous system contributes to the development of depression (Belmaker and Agam, 2008; Kaufman et al., 2016). The 5-HT neurons have both ascending and descending projections (Steinbusch, 1981; Törk, 1990). The ascending fibers project to broad areas of brain, such as forebrain, hippocampus and limbic system (Fischer et al., 1995; El Mansari et al., 2015), suggesting a role that ascending serotonin pathway plays in emotion and cognition. By contrast, the descending 5-HT-ergic fibers contribute more to physiological responses (Jacobs and Azmitia, 1992). There is evidence that spinal nociceptive transmission is modulated by serotonergic fibers descending from raphe nuclei in medulla to spinal cord (Mann and Malone, 1997; Delgado, 2000). In the present study, 5-HT_1A_ receptor antagonism experiment demonstrated involvement of an endogenous analgesia system in pain responsivity and suggested that serotonergic pathways underlying intensity effects on nociception may affect the depression-related modulation of pain. Impairment in monoamine pathways can weaken the supraspinal descending inhibition and thus lead to aggravation of pain (Burke et al., 2010). In classic models of evoked pain, the nociception is always too weak to activate this descending inhibitory system; consequently and theoretically, the stimulus-evoked nociception could not be influenced by depression in the sensory level. However, for spontaneous pain, induction of central sensitization (LaMotte et al., 1991; Tjølsen et al., 1992) and the activation of the descending pain inhibitory system by supraspinal processing (Treede et al., 1992; Urban and Gebhart, 1999; Urban et al., 1999; Schaible et al., 2002) are important phenomena relative to elucidating the interaction of depression and the enhanced pain responsivity.

Given our 5-HT receptor antagonism experimental results, we posit that 5-HT deficiency in depression may influence analgesia efficiency and thereby lead to the transitional modulation of depression (from inhibition to facilitation) on different forms of pain. Our results in animals are consistent with a prior clinical report by Klauenberg et al. (2008), who found that repetitive noxious mechanical stimuli induced increased wind-up in patients with depressive disorder and suggested that a central disinhibition mechanism may correlate with the influence of depression on pain perception. Although the present findings suggest that depression enhances intensive pain but ignores mild pain in the sensory dimension, we still could not explain why depression expressed inhibitory modulation on evoked pain. Furthermore, we tried to find answer from the affective-motivational dimension of pain.

### Reduction in the Pain-Avoidance Motivation Under Depressive Condition

Conditioning paradigms were widely used to evaluate the affective-motivational components of pain (Fan et al., 1995; Shyu et al., 2003). Here, we examined the conditioned avoidance behavior induced by both evoked (tone-laser conditioning) and spontaneous (F-CPA) pain and observed that UCMS-exposed animals exhibited decreased avoidance motivation for evoked pain while their avoidance motivation for ongoing spontaneous pain appeared similar to that of controls. The former results showed the inhibitory effect of depression on evoked pain whereas the latter result suggests that there are combined effects of sensory intensity and affective-motivational level on avoidance of spontaneous pain.

Given that lack of motivation is a core symptom of depression (Nestler and Carlezon, 2006). It may be that reduced responsivity to evoked pain in the depressive condition, which is characterized by a hypo-active 5-HT system, may due to decreased avoidance motivation. Regarding spontaneous pain, it may be that increased pain stimulus delivery augments avoidance motivation in the depressive state, though this effect might be effectively canceled out by the amotivation symptom of depression.

Anhedonia, evidenced by a reduced motivation to pursue rewards, is a core symptom of depression (Mueller et al., 2015; Rizvi et al., 2016). However, the effects of depression on avoidance motivation are unclear and may be complicated (Dickson and MacLeod, 2004; Sherratt and MacLeod, 2013). Fowles have suggested that depression produces decreased approach motivation and increased avoidance motivation (Fowles, 1988). Employing an Iowa Gambling task, Smoski et al. (2008) observed risk aversion of depressed patients suggestive of an increased avoidance motivation for potentially rewarding environmental contexts. Seidel et al. (2010) found that depressed women displayed stronger than typical avoidance tendencies for negative facial expressions. However, Dickson et al. (2011) did not find any differences in avoidance goals between depressed and non-depressed subjects (Dickson and MacLeod, 2004). These findings about approach and avoidance motivation during depression may be related with two primary motivational systems: the behavioral inhibition system (BIS) and the behavioral activation system (BAS). The BIS refers to inhibit ongoing behavior while processing cues of punishment or non-reward and the BAS responds to reward or active avoidance of punishment (Davidson, 1992; Harmon-Jones and Allen, 1997; Balconi and Vanutelli, 2015). Previous studies have mentioned that depression is related with an underactive BAS and overactive BIS (Hundt et al., 2007), which may contribute to the anhedonic symptoms (reduced response to reward) and increased avoidance motivation (inhibited behavior to punishment), respectively.

It should be noted that the meaning of avoidance motivation may not be consistent across contexts (Coan and Allen, 2003). In a majority of psychological studies, avoidance motivation is defined as the desire to maintain a current state, to prevent a negative outcome (Sherratt and MacLeod, 2013), which may correlated with the BIS. However, in the context of pain-avoidance, avoidance motivation may be more related to withdrawal from the pain-related stimulus, which means an active behavior to avoid punishment (the BAS). Therefore, according to the low BAS sensitivity related with depression (Bijttebier et al., 2009; Markarian et al., 2013), it is not a surprise to observe the decreased pain-avoidance behavior in UCMS rats. More importantly, the withdrawal from aversive stimulus may not only involve the behavioral inhibition and activation systems (BIS/BAS), but also the fight/flight system (FFS; Davidson, 1992; Harmon-Jones and Allen, 1997). The FFS responds to aversive stimuli with escape behavior and could be aroused by pain and be arrested by depression (Gilbert and Gilbert, 2003; Carvalho et al., 2013). Our findings suggest that pain-avoidance motivation under depressive state in rats could be related to the fight/flight stress response system being simultaneously aroused by pain-related aversive emotion and inhibited by depression. That is, due to an inhibitory effect of depression on pain-avoidance motivation, avoidance behaviors may be reduced during stimulus-evoked nociception. By contrast, in F-CPA, increased formalin pain responsivity in UCMS group may enhance the desire to avoid the pain-paired context, but the enhanced desire could have been masked behaviorally by depression induced amotivation.

### Limitations

A limitation that stressed animals were not included in the 5-HT experiment should be addressed here. We have the following considerations: (1) Systemic administration of 5-HT_1A_ receptor antagonist in normal rats may mimic the effect of serotonin depletion, as in the depressive condition; and (2) When animal models of depression (i.e., animals of 5-HT deficiency) are involved, further using 5-HT anatogonist in these animals may lead to more complex and confusing results, for example, a ceiling effect may occur, and we may not be able to determine the specific role of 5-HT.

## Conclusion

The present findings elucidating the divergent effects of depression on evoked vs. spontaneous pain indicate that inherent differences among pain models could contribute to seemingly paradoxical phenomena in the literature, namely depression-related inhibition of evoked pain but facilitation of spontaneous/ongoing pain. We demonstrated that the modulatory effect of depression on pain is related to nociceptive stimulus intensity. On the other hand, our pattern of results is consistent with the notion that the decreased motivation associated with depression in the affective-motivational component of pain may lead to a reduction in avoidance behavior. Hence, global nociceptive behavior in a depressive state appears to depend on the superposition of pain intensity and avoidance-motivation.

## Author Contributions

This study was conceived and designed by J-YW and FL; Experiments in this study were performed by NW, S-GL, X-XL, Y-LS, and W-JQ; Data was analyzed by NW and J-YW; This article was written by NW and J-YW.

## Conflict of Interest Statement

The authors declare that the research was conducted in the absence of any commercial or financial relationships that could be construed as a potential conflict of interest.

## References

[B1] Ågren H. Reibring L. (1994). PET studies of presynaptic monoamine metabolism in depressed patients and healthy volunteers. Pharmacopsychiatry 27, 2–6. 10.1055/s-2007-1014265 8159778

[B2] Azpiroz A. Fano E. Garmendia L. Arregi A. Cacho R. Beitia G.. (1999). Effects of chronic mild stress (CMS) and imipramine administration, on spleen mononuclear cell proliferative response, serum corticosterone level and brain norepinephrine content in male mice. Psychoneuroendocrinology 24, 345–361. 10.1016/s0306-4530(98)00084-5 10101738

[B3] Bair M. J. Robinson R. L. Katon W. Kroenke K. (2003). Depression and pain comorbidity: a literature review. Arch. Intern. Med. 163, 2433–2445. 10.1001/archinte.163.20.2433 14609780

[B4] Balconi M. Vanutelli M. E. (2015). Emotions and BIS/BAS components affect brain activity (ERPs and fNIRS) in observing intra-species and inter-species interactions. Brain Imaging Behav. 10, 750–760. 10.1007/s11682-015-9443-z 26319406

[B5] Bär K. J. Brehm S. Boettger M. K. Boettger S. Wagner G. Sauer H. (2005). Pain perception in major depression depends on pain modality. Pain 117, 97–103. 10.1016/j.pain.2005.05.016 16061323

[B6] Belmaker R. H. Agam G. (2008). Major depressive disorder. N. Engl. J. Med. 358, 55–68. 10.1056/NEJMra073096 18172175

[B7] Bijttebier P. Beck I. Claes L. Vandereycken W. (2009). Gray’s reinforcement sensitivity theory as a framework for research on personality-psychopathology associations. Clin. Psychol. Rev. 29, 421–430. 10.1016/j.cpr.2009.04.002 19403216

[B8] Boettger M. K. Grossmann D. Bär K. J. (2013). Thresholds and perception of cold pain, heat pain and the thermal grill illusion in patients with major depressive disorder. Psychosom. Med. 75, 281–287. 10.1097/psy.0b013e3182881a9c 23460720

[B9] Burke N. N. Hayes E. Calpin P. Kerr D. M. Moriarty O. Finn D. P.. (2010). Enhanced nociceptive responding in two rat models of depression is associated with alterations in monoamine levels in discrete brain regions. Neuroscience 171, 1300–1313. 10.1016/j.neuroscience.2010.10.030 20955767

[B10] Cao H. Gao Y. J. Ren W. H. Li T. T. Duan K. Z. Cui Y. H.. (2009). Activation of extracellular signal-regulated kinase in the anterior cingulate cortex contributes to the induction and expression of affective pain. J. Neurosci. 29, 3307–3321. 10.1523/jneurosci.4300-08.2009 19279268 PMC2682784

[B11] Cao H. Zang K. K. Han M. Zhao Z. Q. Wu G. C. Zhang Y. Q. (2014). Inhibition of p38 mitogen-activated protein kinase activation in the rostral anterior cingulate cortex attenuates pain-related negative emotion in rats. Brain Res. Bull. 107, 79–88. 10.1016/j.brainresbull.2014.06.005 25038392

[B12] Carvalho S. Pinto-Gouveia J. Pimentel P. Maia D. Gilbert P. Mota-Pereira J. (2013). Entrapment and defeat perceptions in depressive symptomatology: through an evolutionary approach. Psychiatry 76, 53–67. 10.1521/psyc.2013.76.1.53 23458115

[B13] Coan J. A. Allen J. J. (2003). Frontal EEG asymmetry and the behavioral activation and inhibition systems. Psychophysiology 40, 106–114. 10.1111/1469-8986.00011 12751808

[B14] Coderre T. J. Melzack R. (1992). The contribution of excitatory amino acids to central sensitization and persistent nociception after formalin-induced tissue injury. J. Neurosci. 12, 3665–3670. 1326610 10.1523/JNEUROSCI.12-09-03665.1992 PMC6575737

[B15] Davidson R. J. (1992). Anterior cerebral asymmetry and the nature of emotion. Brain Cogn. 20, 125–151. 10.1016/0278-2626(92)90065-t 1389117

[B16] Delgado P. L. (2000). Depression: the case for a monoamine deficiency. J. Clin. Psychiatry 61, 7–11. 10775018

[B17] Dickson J. MacLeod A. (2004). Anxiety, depression and approach and avoidance goals. Cogn. Emot. 18, 423–430. 10.1080/02699930341000013

[B18] Dickson J. M. Moberly N. J. Kinderman P. (2011). Depressed people are not less motivated by personal goals but are more pessimistic about attaining them. J. Abnorm. Psychol. 120, 975–980. 10.1037/a0023665 21553938

[B19] Dirig D. M. Salami A. Rathbun M. L. Ozaki G. T. Yaksh T. L. (1997). Characterization of variables defining hindpaw withdrawal latency evoked by radiant thermal stimuli. J. Neurosci. Methods 76, 183–191. 10.1016/s0165-0270(97)00097-6 9350970

[B20] El Mansari M. Lecours M. Blier P. (2015). Effects of acute and sustained administration of vortioxetine on the serotonin system in the hippocampus: electrophysiological studies in the rat brain. Psychopharmacology (Berl) 232, 2343–2352. 10.1007/s00213-015-3870-9 25665528 PMC4464372

[B21] Fan R. J. Shyu B. C. Hsiao S. (1995). Analysis of nocifensive behavior induced in rats by CO2 laser pulse stimulation. Physiol. Behav. 57, 1131–1137. 10.1016/0031-9384(94)00372-c 7652034

[B22] Fischer C. Hatzidimitriou G. Wlos J. Katz J. Ricaurte G. (1995). Reorganization of ascending 5-HT axon projections in animals previously exposed to the recreational drug (+/−)3,4-methylenedioxymethamphetamine (MDMA, “ecstasy”). J. Neurosci. 15, 5476–5485. 7643196 10.1523/JNEUROSCI.15-08-05476.1995 PMC6577639

[B23] Fitzgibbon M. Finn D. P. Roche M. (2016). High times for painful blues: the endocannabinoid system in pain-depression comorbidity. Int. J. Neuropsychopharmacol. 19:pyv095. 10.1093/ijnp/pyv095 26342110 PMC4815466

[B24] Fowles D. C. (1988). Psychophysiology and psychopathology: a motivational approach. Psychophysiology 25, 373–391. 10.1111/j.1469-8986.1988.tb01873.x 3051073

[B25] Gambassi G. (2009). Pain and depression: the egg and the chicken story revisited. Arch. Gerontol. Geriatr. 49, 103–112. 10.1016/j.archger.2009.09.018 19836622

[B26] Gilbert P. Gilbert J. (2003). Entrapment and arrested fight and flight in depression: an exploration using focus groups. Psychol. Psychother. 76, 173–188. 10.1348/147608303765951203 12855063

[B27] Graff-Guerrero A. Pellicer F. Mendoza-Espinosa Y. Martínez-Medina P. Romero-Romo J. de la Fuente-Sandoval C. (2008). Cerebral blood flow changes associated with experimental pain stimulation in patients with major depression. J. Affect. Disord. 107, 161–168. 10.1016/j.jad.2007.08.021 17904643

[B28] Han C. S. Pae C. U. (2015). Pain and depression: a neurobiological perspective of their relationship. Psychiatry Investig. 12, 1–8. 10.4306/pi.2015.12.1.1 25670939 PMC4310906

[B29] Hargreaves K. Dubner R. Brown F. Flores C. Joris J. (1988). A new and sensitive method for measuring thermal nociception in cutaneous hyperalgesia. Pain 32, 77–88. 10.1016/0304-3959(88)90026-7 3340425

[B30] Harmon-Jones E. Allen J. J. (1997). Behavioral activation sensitivity and resting frontal EEG asymmetry: covariation of putative indicators related to risk for mood disorders. J. Abnorm. Psychol. 106, 159–163. 10.1037/0021-843x.106.1.159 9103728

[B31] Hundt N. E. Nelson-Gray R. O. Kimbrel N. A. Mitchell J. T. Kwapil T. R. (2007). The interaction of reinforcement sensitivity and life events in the prediction of anhedonic depression and mixed anxiety-depression symptoms. Pers. Individ. Diff. 43, 1001–1012. 10.1016/j.paid.2007.02.021

[B32] Iwata K. Tsuboi Y. Yagi J. Toda K. Furukawa T. Yoshimoto A.. (1994). Effect of interstimulus interval on perceived sensation and intradental nerve activity during thermal tooth stimulation in man. Brain Res. 635, 211–216. 10.1016/0006-8993(94)91441-9 8173957

[B33] Jacobs B. L. Azmitia E. C. (1992). Structure and function of the brain serotonin system. Physiol. Rev. 72, 165–229. 1731370 10.1152/physrev.1992.72.1.165

[B34] Jaracz J. Gattner K. Jaracz K. Górna K. (2016). Unexplained painful physical symptoms in patients with major depressive disorder: prevalence, pathophysiology and management. CNS Drugs 30, 293–304. 10.1007/s40263-016-0328-5 27048351 PMC4839032

[B35] Jiang Z. C. Pan Q. Zheng C. Deng X. F. Wang J. Y. Luo F. (2014). Inactivation of the prelimbic rather than infralimbic cortex impairs acquisition and expression of formalin-induced conditioned place avoidance. Neurosci. Lett. 569, 89–93. 10.1016/j.neulet.2014.03.074 24726402 PMC4382360

[B36] Johansen J. P. Fields H. L. Manning B. H. (2001). The affective component of pain in rodents: direct evidence for a contribution of the anterior cingulate cortex. Proc. Natl. Acad. Sci. U S A 98, 8077–8082. 10.1073/pnas.141218998 11416168 PMC35470

[B37] Kaufman J. DeLorenzo C. Choudhury S. Parsey R. V. (2016). The 5-HT1A receptor in major depressive disorder. Eur. Neuropsychopharmacol. 26, 397–410. 10.1016/j.euroneuro.2015.12.039 26851834 PMC5192019

[B38] Klauenberg S. Maier C. Assion H. J. Hoffmann A. Krumova E. K. Magerl W.. (2008). Depression and changed pain perception: hints for a central disinhibition mechanism. Pain 140, 332–343. 10.1016/j.pain.2008.09.003 18926637

[B39] Kulkarni B. Bentley D. E. Elliott R. Youell P. Watson A. Derbyshire S. W.. (2005). Attention to pain localization and unpleasantness discriminates the functions of the medial and lateral pain systems. Eur. J. Neurosci. 21, 3133–3142. 10.1111/j.1460-9568.2005.04098.x 15978022

[B40] Kundermann B. Hemmeter-Spernal J. Strate P. Gebhardt S. Huber M. T. Krieg J. C.. (2009). Pain sensitivity in major depression and its relationship to central serotoninergic function as reflected by the neuroendocrine response to clomipramine. J. Psychiatr. Res. 43, 1253–1261. 10.1016/j.jpsychires.2009.04.012 19467668

[B41] LaMotte R. H. Shain C. N. Simone D. A. Tsai E. F. (1991). Neurogenic hyperalgesia: psychophysical studies of underlying mechanisms. J. Neurophysiol. 66, 190–211. 1919666 10.1152/jn.1991.66.1.190

[B42] Ledermann K. Jenewein J. Sprott H. Hasler G. Schnyder U. Warnock G.. (2016). Relation of dopamine receptor 2 binding to pain perception in female fibromyalgia patients with and without depression—A [^11^C] raclopride PET-study. Eur. Neuropsychopharmacol. 26, 320–330. 10.1016/j.euroneuro.2015.12.007 26708319

[B43] Li J. X. (2015). Pain and depression comorbidity: a preclinical perspective. Behav. Brain Res. 276, 92–98. 10.1016/j.bbr.2014.04.042 24797835 PMC4216773

[B44] Li S. G. Wang J. Y. Luo F. (2012). Adult-age inflammatory pain experience enhances long-term pain vigilance in rats. PLoS One 7:e36767. 10.1371/journal.pone.0036767 22574223 PMC3344941

[B45] Lumley M. A. Cohen J. L. Borszcz G. S. Cano A. Radcliffe A. M. Porter L. S.. (2011). Pain and emotion: a biopsychosocial review of recent research. J. Clin. Psychol. 67, 942–968. 10.1002/jclp.20816 21647882 PMC3152687

[B46] Mann J. J. Malone K. M. (1997). Cerebrospinal fluid amines and higher-lethality suicide attempts in depressed inpatients. Biol. Psychiatry 41, 162–171. 10.1016/s0006-3223(96)00217-x 9018386

[B47] Markarian S. A. Pickett S. M. Deveson D. F. Kanona B. B. (2013). A model of BIS/BAS sensitivity, emotion regulation difficulties and depression, anxiety and stress symptoms in relation to sleep quality. Psychiatry Res. 210, 281–286. 10.1016/j.psychres.2013.06.004 23845417

[B48] Marsala S. Z. Pistacchi M. Tocco P. Gioulis M. Fabris F. Brigo F.. (2015). Pain perception in major depressive disorder: a neurophysiological case-control study. J. Neurol. Sci. 357, 19–21. 10.1016/j.jns.2015.06.051 26233807

[B49] Mueller E. M. Pechtel P. Cohen A. L. Douglas S. R. Pizzagalli D. A. (2015). Potentiated processing of negative feedback in depression is attenuated by anhedonia. Depress. Anxiety 32, 296–305. 10.1002/da.22338 25620272 PMC4374007

[B50] Navratilova E. Porreca F. (2014). Reward and motivation in pain and pain relief. Nat. Neurosci. 17, 1304–1312. 10.1038/nn.3811 25254980 PMC4301417

[B51] Nestler E. J. Carlezon W. A. Jr. (2006). The mesolimbic dopamine reward circuit in depression. Biol. Psychiatry 59, 1151–1159. 10.1016/j.biopsych.2005.09.018 16566899

[B52] Ohayon M. M. Schatzberg A. F. (2003). Using chronic pain to predict depressive morbidity in the general population. Arch. Gen. Psychiatry 60, 39–47. 10.1001/archpsyc.60.1.39 12511171

[B53] Omote K. Kawamata T. Kawamata M. Namiki A. (1998). Formalin-induced nociception activates a monoaminergic descending inhibitory system. Brain Res. 814, 194–198. 10.1016/s0006-8993(98)01086-5 9838110

[B54] Ossipov M. H. Morimura K. Porreca F. (2014). Descending pain modulation and chronification of pain. Curr. Opin. Support. Palliat. Care 8, 143–151. 10.1097/SPC.0000000000000055 24752199 PMC4301419

[B55] Pignatelli D. Maia M. Castro A. R. da Conceição Magalhães M. Vivier J. Defaye G. (2000). Chronic stress effects on the rat adrenal cortex. Endocr. Res. 26, 537–544. 10.3109/07435800009048567 11196426

[B56] Poole H. White S. Blake C. Murphy P. Bramwell R. (2009). Depression in chronic pain patients: prevalence and measurement. Pain Pract. 9, 173–180. 10.1111/j.1533-2500.2009.00274.x 19298363

[B57] Rizvi S. J. Pizzagalli D. A. Sproule B. A. Kennedy S. H. (2016). Assessing anhedonia in depression: potentials and pitfalls. Neurosci. Biobehav. Rev. 65, 21–35. 10.1016/j.neubiorev.2016.03.004 26959336 PMC4856554

[B58] Schaible H. G. Ebersberger A. Von Banchet G. S. (2002). Mechanisms of pain in arthritis. Ann. N Y Acad. Sci. 966, 343–354. 10.1111/j.1749-6632.2002.tb04234.x 12114291

[B59] Seidel E. M. Habel U. Finkelmeyer A. Schneider F. Gur R. C. Derntl B. (2010). Implicit and explicit behavioral tendencies in male and female depression. Psychiatry Res. 177, 124–130. 10.1016/j.psychres.2010.02.001 20199811

[B60] Sherratt K. A. MacLeod A. K. (2013). Underlying motivation in the approach and avoidance goals of depressed and non-depressed individuals. Cogn. Emot. 27, 1432–1440. 10.1080/02699931.2013.786680 23627339

[B61] Shi M. Qi W. J. Gao G. Wang J. Y. Luo F. (2010a). Increased thermal and mechanical nociceptive thresholds in rats with depressive-like behaviors. Brain Res. 1353, 225–233. 10.1016/j.brainres.2010.07.023 20637742 PMC2933300

[B62] Shi M. Wang J. Y. Luo F. (2010b). Depression shows divergent effects on evoked and spontaneous pain behaviors in rats. J. Pain 11, 219–229. 10.1016/j.jpain.2009.07.002 20096641 PMC2835830

[B63] Shyu B. C. Chai S. C. Kung J. C. Fan R. J. (2003). A quantitative method for assessing of the affective component of the pain: conditioned response associated with CO2 laser-induced nocifensive reaction. Brain Res. Protoc. 12, 1–9. 10.1016/s1385-299x(03)00041-2 12928039

[B64] Smoski M. J. Lynch T. R. Rosenthal M. Z. Cheavens J. S. Chapman A. L. Krishnan R. R. (2008). Decision-making and risk aversion among depressive adults. J. Behav. Ther. Exp. Psychiatry 39, 567–576. 10.1016/j.jbtep.2008.01.004 18342834 PMC2590786

[B65] Steinbusch H. W. (1981). Distribution of serotonin-immunoreactivity in the central nervous system of the rat-cell bodies and terminals. Neuroscience 6, 557–618. 10.1016/0306-4522(81)90146-9 7017455

[B66] Su Y. L. Wang N. Gao G. Wang J. Y. Luo F. (2010). The effect of depression on the thermal nociceptive thresholds in rats with spontaneous pain. Neurosci. Bull. 26, 429–436. 10.1007/s12264-010-0932-1 21113193 PMC4382591

[B67] Tjølsen A. Berge O. G. Hunskaar S. Rosland J. H. Hole K. (1992). The formalin test: an evaluation of the method. Pain 51, 5–17. 10.1016/0304-3959(92)90003-t 1454405

[B68] Törk I. (1990). Anatomy of the serotonergic system. Ann. N Y Acad. Sci. 600, 9–34; discussion 34–35. 10.1111/j.1749-6632.1990.tb16870.x 2252340

[B69] Treadway M. T. Zald D. H. (2011). Reconsidering anhedonia in depression: lessons from translational neuroscience. Neurosci. Biobehav. Rev. 35, 537–555. 10.1016/j.neubiorev.2010.06.006 20603146 PMC3005986

[B70] Treede R. D. Meyer R. A. Raja S. N. Campbell J. N. (1992). Peripheral and central mechanisms of cutaneous hyperalgesia. Prog. Neurobiol. 38, 397–421. 10.1016/0301-0082(92)90027-c 1574584

[B71] Urban M. O. Gebhart G. F. (1999). Supraspinal contributions to hyperalgesia. Proc. Natl. Acad. Sci. U S A 96, 7687–7692. 10.1073/pnas.96.14.7687 10393881 PMC33602

[B72] Urban M. O. Zahn P. K. Gebhart G. F. (1999). Descending facilitatory influences from the rostral medial medulla mediate secondary, but not primary hyperalgesia in the rat. Neuroscience 90, 349–352. 10.1016/s0306-4522(99)00002-0 10215139

[B73] Vidal C. Jacob J. (1986). Hyperalgesia induced by emotional stress in the rat: an experimental animal model of human anxiogenic hyperalgesia. Ann. N Y Acad. Sci. 467, 73–81. 10.1111/j.1749-6632.1986.tb14619.x 3524389

[B74] Wang N. Shi M. Wang J. Y. Luo F. (2013). Brain-network mechanisms underlying the divergent effects of depression on spontaneous versus evoked pain in rats: a multiple single-unit study. Exp. Neurol. 250, 165–175. 10.1016/j.expneurol.2013.09.021 24100021 PMC4386997

[B75] Wang W. Qi W. J. Xu Y. Wang J. Y. Luo F. (2010). The differential effects of depression on evoked and spontaneous pain behaviors in olfactory bulbectomized rats. Neurosci. Lett. 472, 143–147. 10.1016/j.neulet.2010.01.075 20138969 PMC2834830

[B76] Willner P. Towell A. Sampson D. Sophokleous S. Muscat R. (1987). Reduction of sucrose preference by chronic unpredictable mild stress and its restoration by a tricyclic antidepressant. Psychopharmacology (Berl) 93, 358–364. 10.1007/bf00187257 3124165

